# General dentists’ readiness and barriers in intimate partner violence screening: a cross-sectional study in Jeddah City

**DOI:** 10.1186/s12903-022-02627-y

**Published:** 2022-12-09

**Authors:** Ehab N. Alshouibi

**Affiliations:** Department of Dental Public Health, Faculty of Dentistry, King Abdulaziz University, Jeddah, Saudi Arabia

**Keywords:** Intimate partner violence, Domestic violence, General dentists, Screening

## Abstract

**Background:**

Intimate partner violence (IPV) has varying prevalence rates and risk factors based on cultural, social, and economic backgrounds. However, it is common for IPV victims to be seen in dental settings on a regular basis. Identifying IPV victims in dental settings might help address this overlooked global dilemma. This study was conducted to evaluate general dentists’ readiness and potential barriers to performing IPV screening in a dental setting.

**Methods:**

A cross-sectional study was conducted using the Domestic Violence Healthcare Providers Survey (DVHPS). This validated and structured questionnaire was adopted, modified, and self-administered to evaluate general dentists’ readiness and barriers to IPV screening. General dentists were recruited for the study population from 5 administrative territorial regions in Jeddah, Saudi Arabia. The questionnaire consists of 7 domains that assess general dentists’ readiness and barriers to performing IPV screening for all dental patients.

**Results:**

Out of the 273 dentists approached, a total of 200 participated in the study, with a total response rate of 73.3%. The percentages of female and male dentists were 54% and 46%, respectively. Almost 73% of the study population believed they could identify IPV in a dental setting. The multiple linear regression model indicated a substantial decrease in general dentists’ readiness to perform IPV screening due to fear of offending patients, an increased number of patients treated per day, and professional role resistance.

**Conclusion:**

The current study suggests the high readiness of general dentists to perform IPV screening for all patients. However, prominent barriers to implementing IPV screening for all patients include fear of offending patients, an increased number of patients treated per day, and professional role resistance. Postgraduate continuous education is encouraged to re-enforce the importance of IPV screening in dental settings.

**Supplementary Information:**

The online version contains supplementary material available at 10.1186/s12903-022-02627-y.

## Introduction

The head and neck are the most common locations of physical injuries caused by IPV, and therefore, the dental setting can be recognized as an important venue to identify patients who experience IPV [1, 2]. Common specific orofacial signs of abuse identified through dental examination include bruising of the face or neck, bite marks, tearing of the labial frenum, lips and/or mucosal lining, lacerations, nonvital teeth, discolored teeth, traumatic fractured teeth, jaw fractures, and multiple injuries that are in different healing stages [3]. Additionally, behavioral indicators of IPV can be probed in dental settings, which includes a wide range of abusive behaviors, such as dental neglect, failure to attend dental appointments due to perpetrator restrictions, unnecessary partner attendance at appointments, and a patient’s reluctance to speak in the presence of their partner [4, 5]. Former studies have suggested that dental professionals might fail to identify IPV due to a lack of knowledge, training, or referral system, concerns related to the reputation of their practice, consideration for patients’ responses, safety, or privacy, and even litigation against the dental practice by IPV perpetrators [4, 5].

Studies have shown that almost half of IPV victims see a dentist when they have visible signs of abusive physical assaults on their orofacial structures, but less than a fraction of dental health care providers try to identify cases of IPV [4, 6]. On the contrary, other studies have shown that dental care providers play a large role in supporting IPV victims by providing help, showing empathy, and acknowledging their worth [7, 8]. Dentists are in an ideal position to detect IPV and provide victims with support, referrals, and appropriate treatment [7, 8]. However, routine IPV screening is the least common and most difficult practice among dental care providers. This difficulty can be attributed to a variety of reasons, including but not limited to health care provider discomfort, a lack of resources, fear of privacy intrusion, and the limitation of male health care providers due to the perceived gender preference that patients are more likely to discuss IPV with female providers [4, 9]. Due to the lack of dental literature about the definitive role of dentists in preventing IPV, the current study was conducted to improve the comprehension of the role of general dentists in addressing IPV by screening all patients for IPV in the dental setting. The objective of the current study was to identify general dentists’ readiness and potential barriers to perform IPV screening for all dental patients. The rationale of this study was that a general dentist can be one of the first respondents to an IPV victim. Understanding general dentists’ readiness and identifying potential barriers to performing IPV screening for all dental patients might improve the recognition of general dentists as an effective first line of defense against domestic violence.

## Materials and methods

### Study design, setting, and participants

Ethical approval was obtained from The Research Ethics Committee of the Faculty of Dentistry at King Abdulaziz University in Jeddah, Saudi Arabia (Approval Number: 019–16). This current cross-sectional study followed the Strengthening the Reporting of Observational Studies in Epidemiology (STROBE) statement checklist. The eligibility criteria to participate in the study were as follows: general dentists of both sexes, those holding an active license to practice general dentistry, and full-time practitioners working at least 5 days per week. The exclusion criteria were specialized dentists, part-time general dentists, general dentists with limited licenses or limited privileges, and dental interns. Part-time dentists in Jeddah, Saudi Arabia are not a common practice, and they are usually involved in administration duties. Part-time dentists’ patient interaction and exposure is fundamentally less than full-time dentists. The inclusion of part-time dentist from current study population might biase the findings either as less readiness and more barrier due to less clinical exposure or more readiness and less barrier due to fear of clinical exposure stigma. Therefore, to minimize potential bias, part-time general dentists were excluded.

The current study was carried out from November 2020 to May 2021. A validated, structured, and self-administered questionnaire was used to evaluate general dentists’ readiness and barriers to IPV screening. A nonprobability convenience sampling technique was used to recruit the study population. The questionnaire was downloaded on 3 portable devices (iPads) locked with a password, and three calibrated data collectors approached all general dentists working in all 6 major dental hospitals and 10 primary dental health care centers distributed throughout the five districts of Jeddah, Saudi Arabia. Jeddah is a major metropolitan city on the west coast of Saudi Arabia and is divided into five districts based on geographic location: North, South, West, East, and Central.

### Questionnaire design

The questionnaire used in this study was Shortened version of the Domestic Violence Healthcare Providers Survey (DVHPS) adopted and modified from previous studies [5, 10]. It was constructed in English and took approximately 15–20 min to complete. Shortened version of DVHPS is a universal and commonly used assessment tool to identify health care providers’ readiness and impeding barriers to perform domestic violence screening in health care setting [10]. Previous studies indicated an acceptable psychometric evaluation for every item domain with Cronbach alpha ranging from 0.73 to 0.91[11–13]. Also, study showed an averaged overall Cronbach alpha for all items domains of 0.88. Based on all these findings, shortened version of DVHPS is utilized as the most suitable tool to measure the outcome of interest in the current study [11–13].

The questionnaire’s cover page included a detailed explanation of the study’s objective and voluntary nature, anonymity, confidentiality assurance, and contact information of the study investigator for any study-related inquiries. The questionnaire consisted of 29 closed-ended questions in 7 main domains. The first domain consisted of 8 items assessing participants’ demographics and readiness to perform IPV screening. The item used to assess general dentists’ readiness to perform IPV screening were scored using a 10-point scale ranging from 1, which represented the lowest readiness, to 10, which represented the highest readiness. The second domain consisted of 7 items evaluating general dentists’ self-efficacy in performing IPV screening. The third domain comprised 4 items assessing general dentists’ fears of offending patients in performing IPV screening. The fourth domain consisted of 5 items examining general dentists’ judgments of victim personalities and their effect on IPV screening. The fifth domain included 3 items evaluating professional role resistance to perform IPV screening in general dentists. The sixth and seventh domains included 2 items each to explore general dentists’ assessments of victim disobedience and psychiatric support, respectively. Items responses in the second to the seventh domains are recorded on a 5-point Likert scale (1 = strongly disagree, 2 = disagree, 3 = uncertain, 4 = agree, 5 = strongly agree). The predetermined criteria utilized in a previous study were adopted to calculate the mean of each DVHPS domain by creating a summative variable of all items in the domain and dividing by the total number of items in the domain [10].

### Study variables

Descriptive characteristics, including sex (male and female) and whether training in identifying IPV was received in undergraduate education, were dichotomized (Yes/No) variables. Additionally, age, years of dental experience, working hours/week, and the number of treated patients/day were included as descriptive continuous variables. General dentists’ readiness to perform IPV screening was the dependent outcome of interest, and it was measured as a continuous variable using a 10-point scale. Summative variables of DVHPS domains, which included the Self-Efficacy, Fear of Offending Patients, Victim Personality/Trait, Professional Role Resistance, Victim Disobedience, and Psychiatric Support domains, were used in a multiple linear regression model as continuous predictors/confounders of the dependent outcome.

### Statistical analysis

Statistical analyses were conducted using the Statistical Package for the Social Sciences (SPSS) software program, version 22.0 (IBM, Armonk, New York). Descriptive univariate analyses, such as the mean, standard deviation, frequencies and percentages, are used to report the demographic characteristics of the study participants. The means of general dentists’ readiness to perform IPV screening and those of the DVHPS domains were calculated. Collinearity assessment and multiple linear regression model analysis were conducted to evaluate the unconfounded potential association between different predictors in the model with general dentists’ readiness to perform IPV screening as an outcome. Covariates and predictors in the multiple linear regression model were selected from previous literature [10–13]. The significance level was set at *P* < 0.05.

## Results

Out of the 273 dentists approached, a total of 200 participated in the study, with a total response rate of 73.3%. More than half of the dentists were female (54%). Approximately 81% of the study participants received undergraduate education and training in identifying IPV, but only 73% believed they could identify IPV in a dental setting (Table 1). The mean age of the study population was 34.4 years, with an average of 8.6 years of dental experience, an average of 35.3 working hours/week, and an average of 11.1 patients treated per day. The mean readiness score of general dentists for performing IPV screening for all patients was as high as 7.7 (Table 2). A detailed demonstration of the study participants’ responses to each item in each domain of the DVHPS is presented in Table 3. The summative mean variable of each domain in the DVHPS was estimated to facilitate the interpretation of the study population responses (Table 4). Based on previous studies, an estimated mean domain score of ≥ 4 signified a high level of the assessed characteristic of the domain. On the other hand, a mean score < 4 denoted a low level of that domain’s characteristic [10, 11]. Based on these criteria, the results suggested adequate self-efficacy, low judgment for victim personalities/traits, and victim disobedience. In contrast, general dentists showed high professional resistance, high fear of offending patients, and low psychiatric support. These results might imply that general dentists’ professional resistance and high fear of offending patients and a lack of psychiatric support are potential barriers to readiness to perform IPV screening for all patients in dental settings (Table 4).

**Table 1:** Demographic characteristics of the study population

Variable		Percentage
Gender	Male	(92) 46%
	Female	(108) 54%
Received training in identifying IPV in undergraduate education	Yes	(162) 81%
	No	(38) 19%
I believe I can identify IPV in dental setting	Yes	(146) 73%
	No	(54) 27%

**Table 2:** Mean and standard deviation of study participants’ demographics

Variable	Mean ± standard deviation
Age	34.4 ± 5.7
Years of dental experience	8.6 ± 2.1
Working hours/week	35.3 ± 4.4
Number of patients treated/day	11.1 ± 3.6
Readiness to do IPV screening for all patients	7.7 ± 2.2

**Table 3:** General dentists responses to the items in domestic violence healthcare providers Survey (DVHPS) domains

Domain	Strongly disagree	Disagree	Uncertain	Agree	Strongly agree	Total
**1-Self-efficacy**						
I have time to ask about IPV in my practice	(44) 22%	(36) 18%	(4) 2%	(50) 25%	(66) 33%	200
There are strategies I can use to help victims of IPV change their situation	(70) 35%	(60) 30%	(0) 0%	(40) 20%	(30) 15%	
I feel confident that I can make the appropriate referrals for abused patients	(48) 24%	(66) 33%	(6) 3%	(38) 19%	(42) 21%	
I have ready access to information detailing management of IPV	(32)16%	(36) 18%	(0) 0%	(78) 39%	(54) 27%	
I have ready access to medical social workers or community advocates to assist in the management of IPV	(68) 34%	(56) 28%	(8) 4%	(36) 18%	(32) 16%	
I feel that General Dentists can help manage IPV patients	(6) 3%	(12) 6%	(16) 8%	(114) 57%	(52) 26%	
**2-Fear of offending patients**						
I am afraid of offending the patient if I ask about IPV	(40) 20%	(26) 13%	(0) 0%	(46) 23%	(88) 44%	200
Asking patients about IPV is an invasion of their privacy	(44) 22%	(20) 10%	(0) 0%	(42) 21%	(94) 47%	
It is demeaning to patients to question them about abuse	(42) 21%	(24)12%	(10) 5%	(56) 28%	(68) 34%	
If I ask non–abused patients about IPV, they will get very angry	(38) 19%	(44) 22%	(14) 7%	(50) 25%	(54) 27%	
**3-Victim personality/trait**						
A victim must be getting something out of the abusive relationship, or else he/she would leave	(84) 42%	(86) 43%	(20) 10%	(6) 3%	(4) 2%	200
People are only victims if they choose to be	(78) 39%	(84) 42%	(16) 8%	(10) 5%	(12) 6%	
When it comes to domestic violence victimization, it usually takes two	(26) 13%	(38) 19%	(22) 11%	(48) 24%	(66) 33%	
I have patients whose personalities cause them to be abused	(44) 22%	(94) 47%	(14) 7%	(56) 28%	(20) 10%	
The victim’s passive-dependent personality often leads to abuse	(76) 38%	(58) 29%	(8) 4%	(36) 18%	(22) 11%	
**4-Professional role resistance**						
It is not my place to interfere with how a couple chooses to resolve conflicts	(16) 8%	(6) 3%	(0) 0%	(102) 51%	(76) 38%	200
Investigating the cause of IPV is not part of dental practice	(10) 5%	(8) 4%	(4) 2%	(96) 48%	(82) 41%	
If patients do not reveal abuse to me, then they feel it is none of my business	(12) 6%	(10) 5%	(18) 9%	(82) 41%	(78) 39%	
**5-Victim disobedience**						
Women who choose to step out of traditional roles are a major cause of IPV	(88) 44%	(40) 20%	(14) 7%	(50) 25%	(20) 10%	200
The victim has often done something to bring about violence in the relationship	(90) 45%	(42) 21%	(4) 2%	(40) 20%	(24) 12%	
**6-Psychiatric support**						
I have ready access to mental health services should our patients need referrals	(72) 36%	(46) 23%	(22) 11%	(36) 18%	(24) 12%	200
I feel that the mental health services at my clinic or agency can meet the needs to IPV victims in cases where they are needed	(78) 39%	(56) 28%	(16) 8%	(32) 16%	(18) 9%

**Table 4:** Collective mean and standard deviation for each domain in shortened version of the domestic violence healthcare providers survey (DVHPS)

Variable	Mean ± standard deviation
Self-efficacy	4.00 ± 0.98
Fear of offending patients	4.31 ± 1.1
Victim personality/trait	2.52 ± 0.77
Professional role resistance	4.96 ± 0.83
Victim disobedience	2.11 ± 0.58
Psychiatric support	1.99 ± 0.69

Collinearity assessment was performed during the analysis process and is presented in Additional file 1: Table S1. The collinearity statistic tolerance of all continuous variables under investigation was greater than 0.1, which indicated minimal collinearity between the predictors under assessment. Additionally, all variance inflation factor (VIF) values were less than 5, which indicated minimal collinearity between predictors. Based on these findings, minimal collinearity is expected with less variation inflation and lower chances of having underestimated the outcome results. A multiple linear regression model was conducted to investigate potential predictors and barriers that impact general dentists’ readiness to perform IPV screening for all patients. Controlling for the potential confounding effect of all predictors, the regression model showed that male dentists had lower readiness (1.98) to perform IPV screening than female dentists. Dentists who received undergraduate education and training in identifying IPV scored 0.53 units higher for readiness to perform IPV screening than those who did not have undergraduate training. In contrast, the model indicated decreases of 0.18, 0.21, and 0.13 in dentists’ readiness to perform IPV screening with each unit increase in age, years of dental experience, and working hours/week, respectively. Moreover, there was a significant 3-unit decrease in IPV screening readiness for each one-unit increase in the number of patients treated/day. None of these findings were statistically significant, except for the number of patients treated per day, with a p value of 0.04. The regression model also indicated an increase in the readiness to perform IPV screening of 5.49 and 2.66 units with each unit increase in the summative variables of self-efficacy and psychiatric support, respectively. The former variable was statistically significant, with a p value of 0.007, while the latter variable was not statistically significant. There was a substantial decrease of 4.76 units in the readiness to perform IPV screening with each unit increase in general dentists’ fear of offending patients. This finding was statistically significant, with a p value of 0.003. Likewise, there was a notable decrease in IPV screening readiness by 1.72, 2.08, and 1.13 units with each unit increase in general dentists’ victim personality/trait judgment, professional role resistance, and victim disobedience, respectively. However, only the professional role resistance variable was statistically significant (Table 5).

**Table 5:** Multivariate regression model to estimate the effect of predictors as potential barriers on general dentists’ readiness to do IPV screening for all patients

Variables	B estimate	SE	*P* value
Intercept	4.66	1.17	> 0.0001
Gender *(Ref. Female)*	− 1.98	0.79	0.064
Received training in identifying IPV in undergraduate education *(Ref. No education)*	0.53	0.08	0.06
Age	− 0.18	0.01	0.37
Years of dental experience	− 0.21	0.09	0.76
Working hours/week	− 0.13	0.04	0.81
Number of patients treated/day	− 3.00	0.12	0.04*
Self-efficacy	5.49	0.85	0.007*
Fear of offending patients	− 4.76	0.23	0.003*
Victim personality/trait	− 1.72	0.51	0.10
Professional role resistance	− 2.08	0.40	0.048*
Victim disobedience	− 1.13	0.31	0.09
Psychiatric support	2.66	0.62	0.1

*Statistically significant at 0.05 level of significance

## Discussion

The high prevalence and devastating impact that IPV has on individuals and the community mandate training of health care professionals, including the dental workforce, to routinely screen, identify and respond to IPV victims. On average, patients see general dentists biannually for regular checkups; therefore, these patients usually have a more amicable relationship with their general dentists. Therefore, the dental setting provides a safe environment and an ideal opportunity for the detection and prevention of IPV and IPV-related dental injuries. Professional dental IPV screening along with compassionate communication and proper victim referral is a critical preventive response aimed at reducing the occurrence, morbidity, and mortality of IPV [14]. The results of this study indicated high readiness to perform IPV screening among dentists, and this finding is consistent with the results of other studies in the dental literature [5, 15]. However, the current study suggested a differential readiness to perform IPV screening based on sex, in which male dentists showed lower readiness (by 1.98 units) than their female counterparts. This observation is comparable with favorable female-differential remarks suggested by previous studies [5, 16–19].

Due to a variety of barriers, it is unlikely for dentists to screen for IPV or IPV-related injuries, although they reported more training in identifying and treating IPV-related oral maxillofacial injuries [6]. After controlling for all possible confounding predictors in the multiple linear regression model, this study showed a decrease in dentists’ readiness to perform IPV screening with each unit increase in age and years of dental experience. This finding could be explained by the lack of continuous education for IPV and the diminution of dentists’ recollection of IPV screening and identification [5, 9, 10]. Moreover, the number of working hours per week and the number of patients treated per day were identified as limiting factors in dentists’ readiness to perform IPV screening. This can be explained by the fact that the longer the dentists work and the more patients they treat might impede the process of IPV screening due to a lack of time. Our findings are consistent with the literature that reported that only 13% of IPV victims reported actual inquiries by dental care providers about their injuries, which indicates marginal interaction of dentists in identifying IPV victims due to the lack of time in their long and busy schedule [6]. The results of this study indicated three dominant barriers that negatively influence general dentists’ readiness to perform IPV screening for all patients. These barriers were fear of offending patients, the number of patients treated per day, and professional role resistance, in descending order. These limiting factors might lead to uncertainty or failure to perform IPV screening, regardless of dental health professionals’ abuse perceptions [5, 10]. The DVHPS domain-based barriers to IPV screening might suggest a lack of education and continuous training for dentists. Studies have suggested that dental care providers who completed continuous education in IPV training had higher confidence levels and were more likely to screen and refer IPV victims [5, 20]. Additionally, the literature suggests that brief and continuous course education for dental health care providers enhances the preparedness and effectiveness to perform IPV screening without causing further harm to patients [21].

A limitation of the current study is that it was cross-sectional; hence, it does not imply causality. In addition, a nonprobability convenience sampling technique was used, which limits the ability to generalize the results to the general dentist population. This study may be prone to self-selection bias due to the nature of participant recruitment and dependence on self-reported information. Specialized dentists were not included in the study, as the study focused on the general dentist population.

Future research is warranted to assess general dentists’ readiness to perform IPV screening for all patients using a larger sample size and a probability sampling technique. Additionally, further research should evaluate the effectiveness of continuous education for IPV and its effect on general dentists’ preparedness to perform IPV screening.

## Conclusions

This study indicated high self-efficacy and readiness of general dentists to perform IPV screening for all their patients. However, the most prominent barriers to providing IPS screening were fear of offending patients, an increasing number of patients treated per day, and professional role resistance of general dental practitioners. Despite the high readiness of general dentists to perform such preventive screening, these barriers might hinder the pervasiveness of IPV screening in dental settings. Therefore, continuous education to reinforce the importance of IPV screening in the dental setting is highly recommended to empower practicing general dentists to implement IPV screening, identify victims and act appropriately on a legal basis.

## Supplementary Information

**Additional file 1.** Collinearity assessment to evaluate potential correlation between all continuous predictors under investigation. Tolerance greater than 0.1, indicate minimal collinearity between the predictors under assessment. Variance Inflation Factor (VIF) less than 5, indicate minimal collinearity between predictors.

## Data Availability

The datasets generated and analyzed during the current study are not publicly available due to confidentiality agreements. However, they can be made available from the corresponding author upon reasonable request.
