# Supplementary material for: General dentists’ readiness and barriers in intimate partner violence screening: a cross-sectional study in Jeddah City
Source: BMC Oral Health. 2022 Dec 9;22:584. doi: 10.1186/s12903-022-02627-y (PMC9733277; doi:10.1186/s12903-022-02627-y)
Supplement: Supplementary file 1 — Additional file 1. Collinearity assessment to evaluate potential correlation between all continuous predictors under investigation. Tolerance greater than 0.1, indicate minimal collinearity between the predictors under assessment. Variance Inflation Factor (VIF) less than 5, indicate minimal collinearity between predictors. [file 12903_2022_2627_MOESM1_ESM.docx]

**General Dentists’ Readiness and Barriers in Intimate Partner Violence Screening: A Cross Sectional Study in Jeddah City**

**Supplementary Table.1: Multivariate Regression Model Collinearity Assessment of Predictors as Potential Barriers on General Dentists’ Readiness to do IPV Screening for All Patients**

| **Variables** | **B Estimate** | **Standard Error** | **p-value** | **Collinearity Statistic** | |
| --- | --- | --- | --- | --- | --- |
|  |  |  |  | **Tolerance** | **Variance Inflation Factor (VIF)** |
| **Constant** | 11.86 | 2.73 | 0.009 |  |  |
| **Years of dental experience** | -0.33 | 0.1 | 0.88 | 0.631 | 1.585 |
| **Working hours/week** | -0.24 | 0.09 | 0.71 | 0.855 | 1.169 |
| **Number of patients treated/day** | -6.20 | 1.15 | 0.002* | 0.699 | 1.431 |
| **Self-Efficacy** | 7.91 | 2.09 | 0.03* | 0.747 | 1.339 |
| **Fear of Offending Patients** | -8.66 | 1.99 | 0.02* | 0.537 | 1.862 |
| **Victim Personality/Trait** | -3.72 | 0.89 | 0.41 | 0.359 | 2.786 |
| **Professional Role Resistance** | -4.11 | 1.33 | 0.036* | 0.299 | 3.344 |
| **Victim Disobedience** | -2.82 | 0.63 | 0.19 | 0.483 | 2.070 |
| **Psychiatric Support** | 4.19 | 0.75 | 0.21 | 0.901 | 1.109 |

*Statistically significant at 0.05 level of significance

able 1 Knowledge of dental therapists (*denotes correct response)

Statement Yes Don't know No

There is evidence that dental 105* (52.5%) 91 (45.5%) 4 (2%)

therapists can perform high quality work

Therapists can only perform 58 (29%) 68 (34%) 74 * (37%)

operative procedures for children

Therapists must work under the 118 (59%) 48 (24%) 34 * (17%)

direct supervision of a
